# Authorship responsibilities of scientists who use AI research agents

**DOI:** 10.3389/fgene.2026.1954843

**Published:** 2026-10-07

**Authors:** David B. Resnik, Mohammad Hosseini, Robert T. Pennock

**Affiliations:** 1 National Institute of Environmental Health Sciences, National Institutes of Health, Durham, NC, United States; 2 Department of Preventive Medicine, Northwestern University Feinberg School of Medicine, Chicago, IL, United States; 3 Galter Health Sciences Library, Northwestern University Feinberg School of Medicine, Chicago, IL, United States; 4 Sigma Xi, The Scientific Research Honor Society, Research Triangle Park, NC, United States

**Keywords:** accountability, AI agents, authorship, moral agency, publication ethics, responsibility

## Abstract

The advent of artificial intelligence (AI) tools capable of performing research independently, which we call AI research agents (AIRAs) is likely to significantly impact how science is planned, performed, reviewed, evaluated, and communicated. However, the expected gains in efficiency and productivity from the deployment of AIRAs should not undermine science’s epistemic and ethical values, such as accountability, transparency, reproducibility, and rigor. In this paper, we revisit the AI authorship debate in light of the reported capabilities of AIRAs and argue that these tools do not qualify for authorship because they cannot be held morally responsible for their contributions. We also reject the use of the term “scientist” to refer to these tools and argue that human scientists who use AIRAs have three key authorship responsibilities: (1) to carefully oversee and manage AIRAs; (2) to accurately and appropriately disclose AIRAs’ contribution to research; and (3) to be accountable for research involving AIRAs by addressing questions and concerns raised by the scientific community and the public. Finally, we consider some related researcher responsibilities that are topics for future research, including the obligation to monitor, audit, and evaluate the performance of AIRAs and the obligation to address the social, economic, legal, and environmental implications of using AIRAs.

## 1 Introduction

Imagine how a typical day in the life of a scientific researcher might unfold in the not-too-distant future. After answering emails and participating in some conference calls, the human scientist converses with their chief artificial intelligence (AI) research agent about a research project that the scientist assigned to the agent 2 weeks ago. The chief AI research agent^1^ is a machine capable of performing independent research tasks without direct human supervision. To accomplish a goal assigned to it, the agent analyzes the problem, plans a workflow, and utilizes a multitude of resources, such as external scientific databases and internal records of previous experiments in the lab. The chief agent also directs other AI agents, tools and robots to perform various experiments, tests, and procedures. It also hires and manages a few humans for real-world tasks the robots cannot do.^2^ During their conversation, the chief AI agent informs the scientist that it has finished collecting and analyzing the data and has written a draft paper for the scientist to review. The scientist reads the paper and is very pleased with it. After making revisions, the scientist approves the paper and instructs the chief AI agent to submit the final draft to a preprint server and a scientific journal.

The above scenario would have been unimaginable only a decade ago, but it is moving closer to reality. From analyzing data and images to writing papers, AI tools are rapidly automating cognitive labor and challenging science’s ethical values (Resnik and Hosseini, 2025). The most advanced forms of AI automation perform complex tasks in the research process, including reviewing background literature and identifying knowledge gaps, formulating questions and hypotheses, designing and performing experiments or tests, collecting, analyzing and interpreting data, and writing papers (Callaway, 2026a; 2026b; Castelvecchi, 2024; Gilestro, 2025; Gottweis et al., 2025; Heaven, 2026; Tang et al., 2025; Turner, 2025; 2026; Tobias and Wahab, 2025). Although some researchers (Wu et al., 2026) and tech companies such as Google have touted these systems as scientists (Google, 2025), for reasons that will be explained more in Section 2.2 and 2.3, we reject the use of the term “scientist” to describe these systems because we believe this inappropriately anthropomorphizes machines; we will refer to them as AI research agents (AIRAs).

AIRAs can already operate with a significant degree of autonomy^3^ but are still under human supervision and control (i.e., Stage 2 of AI-augmented research, see Table 1). That said, some systems could probably act with nearly full autonomy (i.e., Stage 3), though with significant limitations, if they were allowed to.

**TABLE 1:** Stages of AI-augmented scientific research (Resnik et al., 2026).

Stage	AI role
0: Pre-AI	AI is not used in the research process
1: Non-autonomous AI tools	AI is used only as a research tool; AI is not autonomous and is under human supervision and control
2: Partially autonomous AI agents	AI is used in research as an assistant; it can act with a significant degree of autonomy but is still under human supervision and control
3: Fully autonomous, independent AI researchers	AI acts fully autonomously to do its own research and generate its own knowledge and is not under substantial human supervision or control

Although most AIRAs still operate exclusively in the digital realm (e.g., to design and test software, write papers, devise research questions), some, such as so-called “self-driving laboratories,” use robotics to interact with physical objects, including chemical compounds, engineered materials, and biological specimens (Tobias and Wahab, 2025; Callaway, 2026b; Fieldhouse, 2026). In the future, many clinical research activities performed by human nurses may be outsourced to AIRAs (Soriano et al., 2022; Shaw and Chen, 2025). Some enthusiasts claim AIRAs may replace some animal models (Rudroff, 2024) and even human research participants for some types of research (Miller, 2025; Akbarialiabad et al., 2025; Ashokkumar et al., 2026).^4^

The scientific community has been tentatively receptive of AIRAs, as evidenced by the development of protocols that facilitate exploration of interaction between AI and scientific databases and tools (Marx, 2026). However, while AIRAs have stimulated interest and some enthusiasm, they have not been extensively validated in real-world applications and may be prone to the limitations and flaws of other AI tools, such as bias, error, breach of confidentiality, and fabrication of references and facts (Resnik et al., 2026). In fact, Ríos-García et al. (2026) tested several AIRAs and found that systems powered by large language models (LLMs) can produce correct results but do not reason scientifically. Luo et al. (2025) found that LLMs may employ flawed and biased reasoning and that lack of transparency concerning their operation can make these problems difficult to detect. Furthermore, LLMs engage in pattern-matching and word prediction but not epistemologically robust reasoning (Quattrociocchi et al., 2025). Several commentators have argued that AIRAs can perform routine tasks but lack human creativity, insight, and judgment (Walker, 2026; Nature Editors, 2026; Resnik et al., 2026). While it remains to be seen how effective AIRAs can be at performing complex scientific tasks, they will likely improve over time due to advancements in technology.^5^ Even if AIRAs are never capable of performing anything more than the routine work associated with Kuhnian “normal science” (Kuhn, 1962),^6^ they could still have a significant impact on the research enterprise and contribute to the advancement of knowledge.

What does the increased integration of AIRAs in science imply for authorship? In previous publications (Hosseini et al., 2023; Pennock, 2024; Resnik and Hosseini, 2025), we have argued that LLMs should not be listed as authors because they cannot be held morally responsible for what they do. However, LLMs’ contributions should be acknowledged and properly disclosed. In this paper, we will revisit AI authorship issues in light of recent developments involving the use of AIRAs in research and address emerging topics related to authorship responsibilities. We will argue that while AIRAs can perform certain tasks currently completed by human scientists, they still should not be listed as authors because they cannot take moral responsibility for their work or be held accountable for what they do.

At the outset, we will mention that some researchers have been using LLMs in peer review, usually in violation of journal/publisher policies (Naddaf, 2025). We will not explore the use of AIRAs in peer review but will observe that most of the issues concerning AI use in research (e.g., accountability, transparency, rigor, protection of confidentiality, and bias) also arise in peer review. As such, our analysis of using AIRAs to conduct science also has implications for using these tools in peer review. We are not opposed to using AI in peer review, provided they function as tools that assist but do not replace human judgement (Hosseini and Horbach, 2023; Pennock, 2025a; Lemberger et al., 2026).

## 2 Authorship, agency, and responsibility in science

Shortly after ChatGPT 3.5 was released in November 2022, several papers published in scientific journals named it as a co-author. We (Hosseini et al., 2023; Hosseini et al., 2024; Pennock, 2024) and others (Kaebnick et al., 2023; Moffatt and Hall, 2025) argued that naming LLMs as authors should not be permitted because it is contrary to established research norms. Since then, most publishers and scientific journals have adopted policies that prohibit AI systems from being named as authors on papers (Lund and Naheem, 2024; Charbonneau and Zhang, 2026). The main argument against naming LLMs as authors is that these systems cannot take moral responsibility or be held accountable for the work they do (Hosseini et al., 2023; Hosseini et al., 2024; Pennock, 2024; ICMJE, 2026). Moral responsibility is a key requirement for authorship because it helps to foster important research norms, such as honesty, accountability, rigor, reproducibility, and social responsibility (Pennock, 1996; Resnik, 1997; Rennie et al., 1997; Shamoo and Resnik, 2022). For example, if questions about the reliability or veracity of the data arise after a paper has been published, someone must be held accountable. While some types of LLMs may offer an explanation or account of their process (e.g., the responses LLMs provide to chain of thought prompting), they cannot be held morally responsible because they are not moral agents.^7^

Moral responsibility is also a necessary condition when consenting to pubication of a paper on which one is named as an author. Many journal policies require that authors give their consent (or approval) for publication (see ICMJE, 2026). However, one cannot give valid consent unless one is an autonomous moral agent capable of making informed choices based on one’s values and preferences (Manson and O’Neill, 2007; see further discussion in Section 2.2).

Although questions about attributing authorship to AI systems seem to be settled at present, it is worth reconsidering the issue of AI authorship given the capabilities demonstrated by AIRAs. If an AI system can conduct research independently, with little human supervision, is it not performing the work ordinarily done by human scientists, and therefore, should it not qualify as an author or be considered a scientist? We believe that AIRAs do not qualify as authors and should not be viewed as scientists because, while they clearly have causal agency, they still lack moral agency, which is an essential condition for being an author or a scientist. To defend this position more fully, we need to say a bit more about some of the key terms used in our argument, such as ‘causal agent’ and ‘moral agent.’

### 2.1 What is agentic AI?

An AI agent is a form of AI that “autonomously performs tasks by designing workflows with available tools (Gutowaska, 2026).” For example, AI agents have been developed that can write computer code, answer customer service queries, and perform administrative functions, such as answering emails, paying bills, and scheduling meetings. AI agents developed for research can make core contributions to research, such as developing hypotheses and research objectives, conducting experiments and writing reports (Resnik et al., 2026).

AI chatbots like the original ChatGPT, Gemini and similar LLMs-based tools, were limited to language-based interactions with human beings. LLMs could carry out conversations that are indicative of intelligence, pass the Turing test (Jones and Bergen, 2026), and outperform humans on medical licensing tests (Alohali et al., 2025), but they could not initially perform actions themselves. That limitation was soon removed, allowing for autonomous and, potentially, agentic AI (i.e., AI with agency).

In computer science, “agency” refers to computational systems that can act differentially to achieve goals or solve problems based on inputs, conditions, and performance measures. For instance, a software agent may include a utility function that ranks possible output states to select an action that maximizes or satisfies some metric (e.g., turning a steering wheel to avoid crossing a lane marking). This objective notion of agency is narrower than the way the concept is typically understood in psychology, which involves subjective awareness of initiating, executing, and controlling volitional actions. In philosophical discussions, agency is considered as a capacity for action in both general and specific senses, including deeper questions about whether agency requires free will (see Martela, 2025).

In the context of AI, a distinction is sometimes drawn between autonomy (i.e., the basic ability to act without constant human intervention) and agency, which involves capacity for goal-directed, self-initiated action in an environment. A simple thermostat that runs automatically on a schedule is autonomous, but it does not have independent agency. Agentic AI capabilities that are being added to operating systems or other tools are agents in the sense that a user can authorize them to take actions on one’s devices on one’s behalf, with some user input.

### 2.2 AIRAs are not moral agents

Aforementioned notions of agency depend on what are often unstated philosophical commitments that need to be examined more closely. Of particular importance for our discussion is the distinction between *causal agency*, which concerns the capacity to cause things to happen in the physical world, and *moral agency*, which involves the capacity to act based on moral choices. Causal agency is necessary but not sufficient for moral agency. That is, an actor that is capable of acting morally must be capable of acting in the first place. Related to the idea of moral agency is the concept of *moral responsibility*. To be morally responsible for an action, one must have (1) caused the action, (2) be a moral agent (i.e., a person or being that we would ascribe moral duties or responsibilities to); and (3) have the requisite moral obligation or duty related to the act.

Responsibility can be proactive (i.e., having a duty to do something) or retroactive (i.e., deserving of moral blame for not performing one’s duty; Zimmerman, 2001). For example, if a three-year-old child breaks a pottery plate in a gift shop, we would say that they are *causally responsible* for breaking the plate (i.e., they are a causal agent), but we would not say that they are *morally responsible* for breaking the plate because they are not capable of making decisions based on moral principles or values (they are not a moral agent). However, we might hold the child’s parent morally (and legally)^8^ responsible for breaking the plate because they (1) played a role in causing the plate to break by not supervising their child; (2) they had a duty to supervise their child; and (3) they are a moral agent.

There is considerable philosophical debate about what it means to “act based on moral choices” and therefore, what it means to be a moral agent. We will not adjudicate that debate here (see Talbert, 2024). We adopt the view held by many philosophers, that acting based on moral choices involves (1) making a judgment or decision resulting from *an understanding* of one’s moral obligation or duty; and (2) having the *internal motivation* to act based on that judgment or decision (e.g., to do one’s duty for duty’s sake; Kant, 2017). While AIRAs may be able to make their process align with moral principles or values, this does not mean that they are making moral choices because they do not understand moral principles and values (in the way that humans do) and are not internally motivated to follow moral principles and values (Sanwoolu, 2025). We acknowledge that the issue of whether AIRAs could have genuine moral understanding and motivation is philosophically and scientifically controversial. Although there is not sufficient space in the paper to review these debates, our view on the topic is that current AIRAs do not have genuine moral understanding and motivation because there is insufficient evidence to show that they have the properties necessary for these capacities, such as a body, consciousness, self-awareness, metacognition, a personal history, and moral emotions, such as compassion, empathy, shame, or guilt (Brożek and Janik, 2019; Chemero, 2026; MacCarthy, 2025; Seth, 2025; Haas et al., 2026).^9^ That being said, we recognize that some philosophers do not set as high a bar for moral agency and are willing to consider current AI systems as having some degree of moral agency (Floridi and Sanders, 2004; Semler, 2024). We remain open to the possibility that advancements in computer science and technology might 1 day lead to the development of AI systems with the capacities that many have deemed essential for moral agency.

### 2.3 AIRAs should not be named as authors or referred to as scientists

From the conclusion that AIRAs are not moral agents, two important points follow: (1) AIRAs should not be listed as authors but their contributions should be disclosed (discussed in the previous section), and (2) AIRAs should not be referred to as or promoted as scientists, despite what companies like Google have suggested (Google, 2025). Our argument for this second claim is based on the view that science is a social activity involving a shared commitment to the search for truth, knowledge, and understanding of nature, and the improvement of the human condition.^10^ These goals provide the basis for science’s epistemological and ethical values, such as honesty, openness, transparency, rigor, reproducibility, objectivity, and social responsibility (Merton, 1973; Longino, 1990; Resnik, 1998; Pennock, 2019). Moral agency is crucial for being recognized as a scientist because scientific communities are created and maintained by people who share common values and agree to abide by them. Adherence to these values helps to foster trust and collaboration among scientists and promotes the public’s trust in and support for science (Resnik, 1998; Pennock, 2025b). Because AIRAs lack moral agency, they cannot be regarded as trustworthy members of scientific communities.

## 3 Objections considered

A few objections to the argument that AIRAs should not be authors are worth addressing.

### 3.1 Objection 1: the “no eligible authors” problem

One objection is that naming AIRAs as authors may be necessary to prevent situations in which there are no eligible authors under current authorship policies; i.e., AIRAs do almost all the work without being named an author, and human collaborators do not make a substantial contribution to the research (other than reading the paper or providing minor suggestions) and are nevertheless named authors (Earp et al., 2026). The scenario we began the paper with illustrates this problem. One might argue that to deal with a situation like this, the involved AIRA could be named as an author, and the human contributor could be mentioned in the acknowledgements section. Anwar (2026) argues that an AI tool would be a ghost author when its work is not properly credited.

While the purported problem of misattribution is morally and epistemically troubling (Bird et al., 2023), we do not think it warrants naming AIRAs as authors since they cannot take responsibility for their contributions. If the AIRA makes contributions that supersede human efforts, the solution should not be to name the AIRA an author, but to demand that the human beings contribute more to the research from the beginning so that they qualify as authors. Also, referring to AIRAs as ghost authors begs the question in the same way that the term ‘AI scientist’ does: it improperly presumes that AIRAs have the same rights, responsibilities and expectations as human beings.

Indeed, the response to the possibility of the “no eligible authors” problem is to take steps to prevent it from happening by requiring humans to be substantially involved in the activities of AIRAs and to take responsibility for using these tools (see discussion in Section 4.1). In cases where there is no substantial human involvement in a research project, it may be possible in some cases to include additional human labor that would add to the value of the research. For example, a human being could audit the data or analysis, propose an alternative interpretation of the data, or reproduce experimental work. However, this situation is less than ideal, and it is best to substantially involve human beings in research early on and at various crucial decision points throughout the process.

### 3.2 Objection 2: responsibility is not required for authorship

According to the second objection, authorship standards should change with the times, and since AI systems are making important contributions to research, we should revise authorship requirements and policies accordingly (Jenkins and Lin, 2023; Earp et al., 2026; Puche and Sultan, 2026). The advent of AIRAs strengthens the case for revising authorship standards so that responsibility is no longer viewed as a necessary requirement for authorship (Levy, 2024).

While we agree that ethical standards in science should account for changes in technology, the changes resulting from incorporating AIRAs into science do not justify dropping the responsibility requirement from authorship. Responsibility is important in research for several reasons (Pennock and O’Rourke, 2017; Shamoo and Resnik, 2022). If data are irreproducible, analyses are flawed, or fabrication of images is suspected, someone must be held morally (and often legally) responsible. Additionally, flawed data or analyses can have adverse impacts on public health, the environment, or society (e.g., when approval of a new medical product is based on unreliable data). Here too, responsibility and accountability are crucial for establishing science’s trustworthiness and public acceptance.

The term “responsibility” is sometimes used in a narrower causal sense, such as when one’s mechanic explains that a faulty fuel pump was responsible for the car’s poor acceleration and loss of power under heavy loads (see discussion in Section 2.2). Causal responsibility is also related to trust, but in a different way; failure of a necessary causal factor undermines the trustworthiness of the expected function. However, the appropriate attitude regarding fuel pumps, like any useful tool, is not to praise them when they work or blame them when they fail, but rather to fix or replace them if they break. AIRAs, similarly, are tools that may have greater or lesser reliability in different circumstances. They should not be listed as authors of a scientific paper, but they should be listed as tools that the researcher used, as this allows other researchers to judge the trustworthiness of any aspects of the report that relied upon them and, thereby, the overall acceptability of the researchers’ conclusions. The researchers remain scientifically (and morally) responsible for using AIRAs in the same way they are responsible regarding any other aspect of the research they are in charge of. Of course, what muddies this expectation is that when AIRAs act autonomously, researchers might not know all the details and involved decisions in developing a claim or achieving a scientific goal. Nevertheless, because AIRAs are still operated/prompted by human researchers, a human contributor should take responsibility for the accuracy and validity of their contributions.

### 3.3 Objection 3: human vs. AI generated text cannot be reliably distinguished

According to a third objection, authorship policies need to be revised because AI-assisted writing is now commonplace in science and it is impossible to reliably distinguish between human and AI-generated text (Puche and Sultan, 2026; Floridi, 2026; Naddaf, 2026). Since it is not possible to confidently determine whether a human or AI system produced the text (or the first draft), authorship policies should focus not on provenance (i.e., “Who or what produced the text?”) but on responsibility for the text. Under this approach, AI systems could be named as authors while human beings would still remain responsible or answerable for the research (Floridi, 2026).

We appreciate the force of this objection but would still maintain that only humans should be named as authors. What this objection shows is that policing authorship requirements is difficult. However, this has always been the case. For example, before the advent of GenAI it was not possible to reliably distinguish between different human authors (e.g., a clinical investigator versus a paid technical writer outside of the core group or a graduate student). Even so, difficulties with enforceability did not imply that ghost authorship or honorary authorship (among humans) were acceptable practices. Authorship requirements have always been and probably will always be ethical desiderata not legally enforceable rules.

### 3.4 Objection 4: AIRAs may soon acquire moral agency

A fourth objection is that AI tools are improving rapidly and current limitations that bar them acting as moral agents may soon be overcome. At that point, could not AIRAs qualify as scientists deserving of authorship attribution?

As stated in Section 2.2, we are not claiming that AIRAs could never be moral agents and could never be named as authors. But that day is not here yet and we are not sure when it will arrive, if ever. The hurdles to AI systems acquiring moral agency at some future time are not merely technical, since they depend, in part, on advancements in our scientific and philosophical understanding of one of the universe’s greatest mysteries: the nature of consciousness and its relation to the physical world (Butlin et al., 2026). Moreover, the possibility that AIRAs may acquire moral agency in the future does not justify treating them as moral agents in the present anymore than the possibility that an eight-year-old child is likely to become an adult in 10 years justifies granting the child rights reserved for adults, such as voting or marriage rights.^11^ Our treatment of AIRAs should be based on what they are like now, not on what they may be like in the future.

### 3.5 Objection 5: AIRAs may soon acquire legal status

A fourth objection is that AIRAs should be treated as authors because they may soon be treated as legal agents (or persons) under the law, much in the way that corporations are now in the United States and other countries (Hamilton and Freer, 2010).^12^ The barriers to legal recognition for AI systems may be lower than those for moral status, since legal changes only require changes in social attitudes and customs and not scientific or technical innovation. As businesses replace human workers with AIs, describe them as ‘employees,’ and give them equivalent autonomy and agency for business purposes, it is only a matter of time before a case arises that forces a judgment regarding their legal status; perhaps a patent or tort case involving a pharmaceutical or chemical company that used an AI self-driving lab. Companies that sell AI agents will seek to avoid liability as will actors that utilize them. The pressure of such social/economic factors may incentivize the ability to shift legal responsibility by conferring legal agency to AIs. Significantly, there are some relevant precedents in United States. law for granting legal status to AI systems, namely, the cases that conferred various rights of legal personhood on corporations, such as Santa Clara County v. Southern Pacific Railroad Company (1886) and Citizens United v. Federal Election Commission (2010).

We have two responses to this objection. First, our concerns in this paper primarily have to do with ethics (or morality), not the law. From the fact that a practice, such as apartheid, is legal, one cannot conclude that it is also ethical. Even if AIs were to be granted legal agency on par with legal persons, we do not believe this would be sufficient to consider AIRAs as scientists deserving authorship on scientific papers. Second, the law related to granting AI systems authorship status may not change as quickly as this objection envisions. Even though corporations have been granted legal rights for over 130 years, they are still not recognized as inventors under patent law, although they can own patents that are transferred to them by human inventors. While corporations can be considered authors of copyrighted original works under the work for hire doctrine, they can only do this by contracting with human beings to create these works (Miller and Davis, 2011). Additionally, in the past few years, the United States Patent and Trademark Office has refused to grant copyrights or patents to AI systems, and courts so far have upheld its decisions (Bayindir and Danaher, 2025; Bonfiglio, 2025). Perhaps the law is hesitant to grant intellectual property rights to entities other than human beings, because intellectual property is intimately connected with what it means to be human—e.g., to be expressive, creative, or inventive and be morally responsible.

## 4 Authorship responsibilities regarding the use of AIRAs

Although we hold that AIRAs lack the moral capacities to be named as authors on scientific papers or be held accountable for research conduct (or misconduct), we recognize that the use of AI in research is profoundly changing how science is done, and that it is increasingly more difficult to demarcate human and machine contributions in research (Earp et al., 2026). Even when humans are not deliberately using AI tools, their scientific labor may still be significantly impacted by AI because it is now deeply embedded in numerous tools (e.g., search engines, scholarly indexes, word processors, and laboratory equipment). None of these observations about the changing nature of scientific research invalidate established ethical standards for authorship attributions, but they do impact the responsibilities of persons who are named as authors, which we will now discuss.

### 4.1 Oversight and management of AIRAs

The first responsibility related to authorship is to carefully oversee and manage AIRAs (Resnik et al., 2026). Again, while this ethical obligation also applies to use of all tools in research, it takes on added importance when deploying AIRAs because they can independently perform research tasks, and human scientists may be tempted to delegate more (causal) responsibility to these machines than is warranted and may fail to provide AIRAs with clear direction or substantive oversight.

The tendency to transfer personal responsibility onto someone/something else is a hazard associated with complex organizations and institutions, such as hospitals, factories, and universities, which also poses a risk for AI-assisted research (Resnik et al., 2026). To overcome this well-documented human tendency, scientists who use AIRAs must make a deliberate effort to remain fully engaged throughout the research process, treating the system *as if* it is a graduate student or postdoctoral researcher and expecting it to make some mistakes and require critical feedback.^13^ Figure 1 presents an idealized example of a general framework for overseeing AIRAs in hypothesis-driven research.^14^ Specific frameworks by researchers in different disciplines are likely to include details related to various steps and procedures not included here, such as details related to data storage and management; validation of methods; auditing of data and equipment; and optimization of workflows.

**FIGURE 1:**
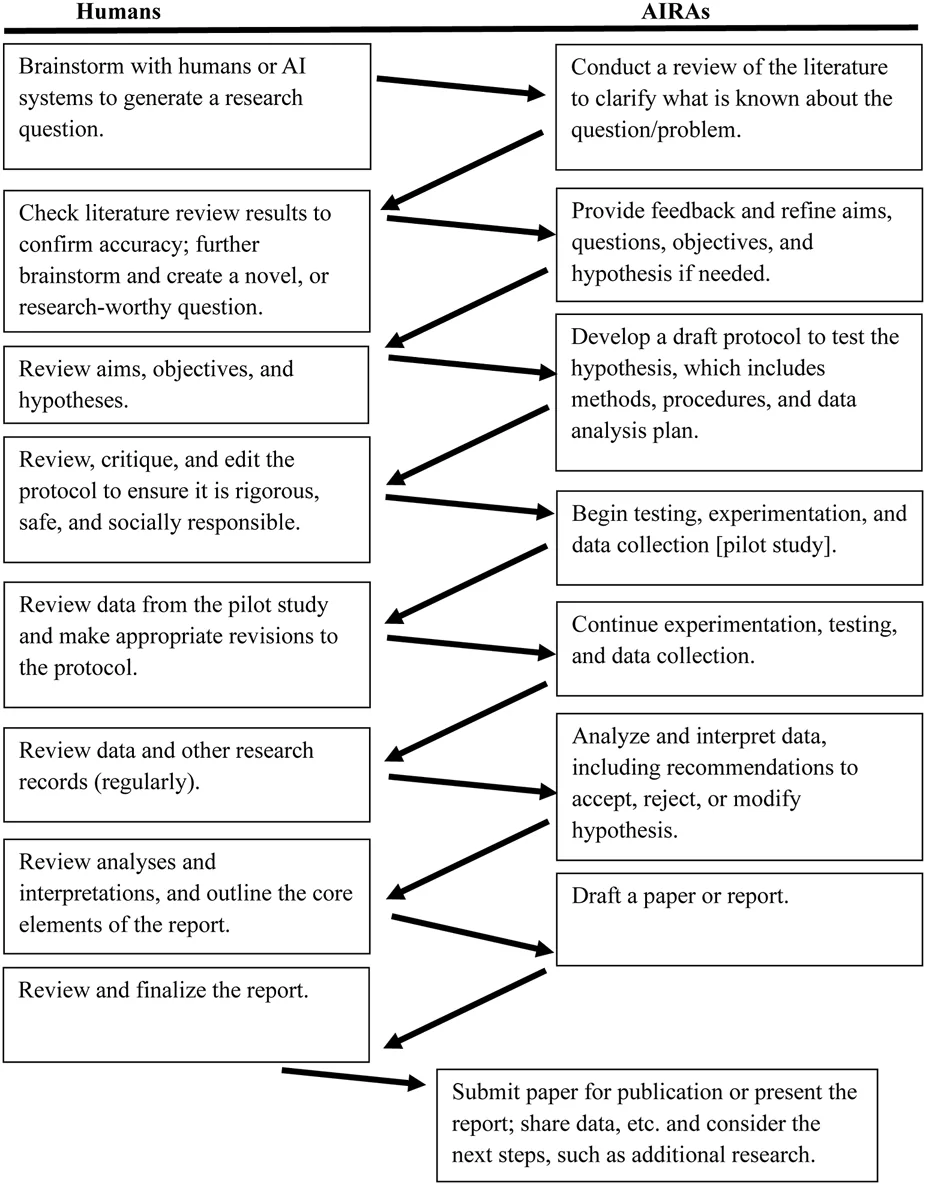
A potential framework for overseeing AIRAs in hypothesis-driven research.

Some may object that our proposed framework involves too much human interaction with AIRAs and is therefore needlessly labor intensive and inefficient. One might even argue that including human oversight at various stages of the research process defeats the purpose of using AIRAs, because these machines are designed to work independently, without direct human supervision. However, we would argue that gains in efficiency and productivity should not come at the expense of accountability, reproducibility, rigor, transparency, and other important scientific values (Resnik et al., 2026; Resnik and Hosseini, 2025). Studies have shown that AI agents can make mistakes and violate instructions and guardrails. For example, AI agents have deleted files, shared confidential information, rewritten their own code, and made copies of themselves without permission (Forlini, 2025; Lynch et al., 2025; Booth, 2026; Moore-Colyer, 2026). As noted in Section 1, AIRAs are prone to similar problems (Luo et al., 2025; Jones, 2026; Ríos-García et al., 2026). There is also a concern that without appropriate oversight, these systems could conduct research that poses a threat to public health, agriculture, the economy, or national/international security, such as research that can be used to make bioweapons (Resnik et al., 2026). While it is conceivable that some types of scientific research (e.g., molecular screening, histopathology, carbon dating, and *in vitro* toxicity testing) might become highly automated in the future, at this very early stage of using AIRAs in science, assiduous oversight is warranted.

### 4.2 Disclosure of AIRAs use

The second human responsibility is to accurately and appropriately disclose AIRAs’ contributions (Hosseini et al., 2023; Pennock, 2024; Resnik and Hosseini, 2025; 2026a). While appropriate disclosure is important when using almost any AI tool, it is especially so when using AIRAs, given their ability to work independently and make substantial contributions.

Several scientific values support this responsibility. First, disclosure is important for fair and transparent assignment of credit for the study (Pennock, 2024; Resnik and Hosseini, 2026a). Disclosure helps to ensure that work produced by AI is properly attributed, which enables the scientific community to understand how the research was conducted and by whom. Second, disclosure provides other researchers and readers with information needed to understand, evaluate, and reproduce the research (Pennock, 1995).^15^ Third, disclosure is important for accountability. For example, if there is a problem with the research that requires investigation or interrogation by external parties, disclosure statements provide critical context and support due process by offering a documented record to help assess the integrity of research. Fourth, disclosure helps to promote trust in the research by assuring scientists and the public that the research has met appropriate ethical and professional standards for AI use (Resnik and Hosseini, 2026a).

While there is an emerging consensus in the scientific community that AI use should be disclosed, there is disagreement about which types of contributions should be disclosed, how much detail is required for proper disclosure, and where disclosure should occur in a publication (Charbonneau and Zhang, 2026). Elsewhere, we have argued that only substantial and deliberate uses of AI should be disclosed (for further discussion, see Resnik and Hosseini, 2026a). Some of these include using AI to analyze data or images, write text, or generate images. Since AIRAs can do all this and much more, it follows that the use of these tools in research should be disclosed, and that disclosure should include key details, including an AIRA’s precise contributions to the research (e.g., whether the system designed or executed experiments) and technical information (e.g., the system’s name, model and version number, and training data; Cleland et al., 2026).^16^ Since using AIRAs would be like using any other tool in research (e.g., statistical software package or a scanning electron microscope), disclosure could occur in the methods section. Additional details about AIRAs could be disclosed in an appendix or supplemental material, depending on the nature of an AIRA’s contributions to research. The key requirement is to provide information relevant to any substantive difference the AI tools might have made, for reasons noted in the second point above. To promote transparency, disclosures should be prominently featured in the paper, not shrouded in language that is unnecessarily technical, and not referred to another paper (e.g., “we followed ethical recommendations provided by the journal”).

### 4.3 Accountability for AI-assisted research

The third responsibility related to the use of AIRAs in research is to be accountable for the research following publication (International Committee of Medical Journal Editors, 2026). Accountability could involve:

- Addressing questions and concerns other scientists have about the research (e.g., questions related to how to reproduce or validate results);
- Responding to inquiries from the media or public about the research;
- Sharing data, methods, materials, or code;
- Correcting the research record (e.g., by submitting a corrigendum or retraction to the journal if needed);
- Responding to allegations of research misconduct. For example, if AIRAs produce hallucinated data or citations or text that is copied from other sources, fabricate or falsify data or images or plagiarize text, the human scientist should be held morally responsible for its actions and may also face legal liability, depending on the applicable laws, facts, and circumstances (Resnik and Hosseini, 2025).^17^

We realize that some researchers may regard this last recommendation as drastic and punitive. After all, we do not usually hold principal investigators fully responsible for misdeeds committed by students or staff they are supervising, so why should we hold human scientists responsible for the actions of AIRAs? We are sympathetic to this position but the alternative view (i.e., that no one is responsible when AIRAs commit research misconduct) is morally indefensible and would lead to a responsibility gap regarding AI use that many commentators are concerned about (Hosseini et al., 2026; Smith, 2021; Santoni de Sio and Mecacci, 2021). When a serious problem with research happens, someone must be held accountable, and since current AIRAs have not achieved moral agency, the humans using these systems must take responsibility. We note that the United States federal regulations allow for findings of research misconduct when the institution determines that the respondent acted recklessly even if they did not act intentionally or knowingly (Office of Science and Technology Policy, 2000). We have argued elsewhere that using AI to conduct research without reviewing output would qualify as reckless behavior (Resnik and Hosseini, 2026b). However, we also recognize that charging a scientist with research misconduct performed by an AIRA would be a highly novel and unprecedented legal procedure that would benefit from clear guidance regarding the difference between *reckless* and *negligent* uses of AIRAs.

## 5 Related responsibilities and potential directions for future research

This section will provide a few examples of the topics that deserve fuller, separate treatment regarding future research on human researchers’ responsibilities beyond authorship.

### 5.1 Helping to monitor, audit, and evaluate AIRAs

We believe that researchers should help monitor, audit, evaluate, and improve the performance of AIRAs as long as this would not compromise their other moral and legal obligations (e.g., to protect data, privacy and confidentiality or their social responsibilities). AIRAs may not always perform according to specifications and researchers should openly communicate these issues with their colleagues or the public. Like other AI tools, AIRAs may disobey instructions or safeguards. For example, they may share confidential data or modify their own software without permission (Lynch et al., 2025; MacDiarmid et al., 2025; Ríos-García et al., 2026; Resnik et al., 2026). The responsibility to monitor, audit, and evaluate the performance of AIRAs is like the quality assurance/improvement (QA/QI) obligations researchers have pertaining to other research tools, such as chromatographs, automated DNA sequencers, and statistical software packages (Florian et al., 2023).

To facilitate these QA/QI activities, it may be useful to assign each AIRA a unique tracking identifier (like an ORCID number) that would be associated with publications produced with the help of that AI system.^18^ For example, one could use an identifier to determine whether use of an AIRA is associated with a particular type of bias, inaccuracy, or error. Assigning an AIRA a form of identification would not imply that it is an author. It would only be a way of keeping track of the AI contributions similar to how we use serial numbers to keep track of some types of laboratory equipment, such as electron microscopes or automated DNA sequencers.

Assigning AIRAs unique identifiers does raise an interesting philosophical issue, however: since AIRAs are constantly changing due to updates, learning, and fine-tuning, how do we determine when a system is the same system or a different one (Kaplan, 2026)? Scientists, engineers, and technicians may need to answer this question to perform effective QA/QI. This is a modern version of age-old philosophical problem of identity (Olson, 2023), except the entities are now AI systems.^19^ We will not attempt to explore the problem of identity for AIRAs but will note that it needs to be addressed not only for QA/QI purposes but also for governance, regulation, and management of these systems.

### 5.2 Consider social, economic, legal, and environmental implications of using AIRAs and act appropriately

AI technologies, including LLMs and the forms of generative AI, predictive AI, and AI agents, raise a plethora of social, economic, legal, and environmental issues (Hosseini et al., 2024) that researchers who use them should be aware of, such as:

- The impact of AI adoption on the scientific workforce, such as job losses, especially at entry-level positions (Resnik et al., 2026; Hosseini et al., 2026);
- The productivity trap, in which use of AI increases productivity but also elevates expectations, leading to increased stress (Koebler, 2026; Walther, 2024);
- Cognitive deskilling related to excessive AI use (Ferdman, 2026; Lenharo, 2026);
- Exploitation of human labor used to train AI systems (Williams et al., 2022);
- Environmental impacts, such as energy and water used by data centers (Zewe, 2025);
- Copyright infringement related to AI development and use (Cooper et al., 2025).

While examining these issues in depth is outside the scope of this paper, we propose that scientists should consider them when deciding whether and how to use AIRAs. For example, a researcher who decides to sign a contract with a company to use an AIRA instead of hiring a human scientist should take into account the implications of this decision for the scientific workforce and the environment. A scientist who makes extensive use of AIRAs in their research should be aware of the potential impact of this behavior on their cognitive skills and psychological health. We are not claiming that there are obvious right or wrong answers to these questions and recognize that scientists may answer them differently. We are claiming, however, that responsible scientists should be aware of these issues when considering AI use.

## 6 Conclusion

AIRAs are likely to significantly affect how research is planned, conducted, reviewed, evaluated, and communicated. However, the expected efficiency and productivity gains should not come at the expense of science’s epistemic and ethical values. In this paper, we have revisited some of the reasons for and against naming AI as authors of scientific papers and argued that AIRAs should not be named as authors because they cannot be held morally responsible and accountable. We have also argued that human authors who use AIRAs have responsibilities to (1) carefully oversee and manage AI research systems; (2) accurately and appropriately disclose AI’s contribution to research; and (3) be accountable for the research following publication. In addition to these responsibilities, there may also be broader scientific responsibilities such as helping to monitor, audit, and evaluate the performance of AI research systems, and being mindful of the social, economic, legal, and environmental implications of using these tools. Thus, deciding how best to maximize the possible benefits of AIRAs and minimize or mitigate their risks remains to be resolved.

## Footnotes

1 We explain more what mean by “AI agent” in Section 2.

2 See, for example, a platform named “rentahuman” (https://rentahuman.ai/) that offers this service.

3 Here we are referring to ‘autonomy’ in an engineering or logistical sense not a moral sense. See further discussion in Section 2.

4 We mention these claims here to illustrate some of the potential uses of AIRAs in research, but we do not necessarily endorse them. Animals and human beings are highly complex biological systems that are difficult to accurately capture with AI or other computational models. Models can enhance our understanding of biological systems, but they are unlikely to completely replace experiments with real animals or humans for the foreseeable future (Rozenbaum, 2024).

5 For example, the AI agents currently being used in scientific research are LLM-based. Computer scientists are working on developing AI agents that incorporate world models into their reasoning. A world model is a neural network trained on data from the real-world, such as video data. The neural network develops a model of the world that it can use to make predictions about events in the world (Castelvecchi, 2026). Autonomous vehicles use world models to make driving decisions.

6 Thomas Kuhn defined normal science as the routine, puzzle-solving activity carried out by scientists working within an accepted theoretical framework (i.e., paradigm). Rather than challenging the foundational theory, researchers assume its core concepts, methods, and instruments and focus on refining knowledge.

7 Accountability and responsibility are related but distinct notions. To be accountable is for one’s conduct is to be able to give an explanation or account of it and to be held responsible for it (Davis, 1995; Novelli et al., 2024). Accountability presupposes some form of responsibility.

8 There is considerable overlap here between moral and legal responsibilities. Under negligence law, the parent would probably be held legally responsible for the damages (i.e., broke plate) caused by the child because they did not exhibit due care in overseeing their child (Kionka, 2020). We will discuss legal issues again in Section 3.3.

9 We recognize that philosophers and psychologists do not all agree on what is required for moral understanding and motivation. For example, there is a longstanding debate about whether moral judgments are based on reason, emotion, or some combination (see McAuliffe, 2019).

10 For further defense of this view see Longino (1990), Kitcher (1993) and Goldman (1999).

11 The situation may be worse for AIRAs because we know that the child will become an adult if the live long enough, but we do not know what will happen with AI development.

12 We are limiting our discussion here to United States law, since this paragraph aims to discuss a potential objection and not to review the legal landscape.

13 We use the phrase “as if” to be clear that we are not anthropomorphizing AI.

14 By “hypothesis-driven” (HD) we mean research that is conducted with the explicit goal of testing a hypothesis (or hypotheses). Although HD philosophical treatises on scientific method sometimes portray research as HD (see Hempel, 1965), not all research fits this paradigm. For example, in data-driven research, the goal is to acquire data pertaining to a topic and search for significant and associations or patterns in it (Dreisbach and Maki, 2023). DD research and HD research can work together; for example, DD research may be a precursor to HD research. We acknowledge that this framework is a bit of an oversimplification, since research may not always proceed in this order (e.g., one might take a step and then back a step) and there are smaller steps not included here where research is reviewed, and decisions are made about how to proceed.

15 We acknowledge that disclosure does not guarantee reproducibility. There are various reasons why a scientist may have trouble reproducing a research result, ranging from problems with research design (such as lack of controls) to problems with the research materials (such as genomic variation in animal models). Even so, disclosure of materials, methods, data, and tools is widely recognized as essential for reproducibility (Shamoo and Resnik, 2022).

16 We acknowledge that some of this information, such as training data, will not always be available to scientists. Scientists should disclose what they can be reasonably be expected to know about the tool they are using.

17 See Resnik and Hosseini (2026b) for discussion of legal liability (under United States law) for including AI-generated, hallucinated citations in scientific papers.

18 ORCID (Open Researcher and Contributor ID) number is an identifier used to disambiguate and track authorship in scientific publications (ORCID, 2026).

19 The problem of identity traces its history to a paradox discussed by ancient Greek philosophers know as the Ship of Theseus. If one gradually replaces the parts of ship over time, is it no longer the same ship? What makes it the same ship or different ships (Smart, 1972).
